# Successful treatment of extensive-stage small cell lung cancer with concurrent pleural and pericardial effusions: Case report

**DOI:** 10.3389/fonc.2022.1040452

**Published:** 2022-12-23

**Authors:** Ayaka Kashima, Yosuke Fukuda, Miri Shimamura, Miharu Ijichi, Hironori Sagara

**Affiliations:** Department of Medicine, Division of Respiratory Medicine and Allergology, School of Medicine, Showa Universityo, Tokyo, Japan

**Keywords:** pericardial effusion, pleural effusion, pleurodesis, small-cell lung cancer, immune checkpoint inhibitor

## Abstract

It is unclear whether pleural/pericardial drainage and pleurodesis/pericardiodesis should be performed before or after initiating chemotherapy in patients with chemotherapy-sensitive small-cell lung cancer. A 76-year-old woman presented to the emergency department with progressive dyspnea on exertion for a week. Chest computed tomography showed a mass shadow anterior to the left upper lobe, bilateral pleural effusions, and a circumferential pericardial effusion surrounding the heart. We diagnosed extensive-stage small-cell lung cancer based on the clinical course and pathological findings. We first performed pleurodesis and pericardial drainage and successfully initiated immune checkpoint inhibitor combined chemotherapy, with improved performance status. This case highlights the importance of aggressive drainage and pleurodesis/pericardiodesis, and suggests that drainage and pleurodesis/pericardiodesis should be considered before systemic chemotherapy in patients with concurrent pericardial or pleural effusions, even in patients with small-cell lung cancer that is sensitive to chemotherapy.

## Introduction

Small-cell lung cancer (SCLC) is rapidly progressive and is highly sensitive to chemotherapy compared to non-small-cell lung cancer. SCLC with concurrent pleural and pericardial effusions at the time of the initial presentation is rare. A retrospective observational study found that of 765 patients with SCLC, 63 had pleural effusions, 17 had pericardial effusions, and 16 had both (1). Recently, the initiation of chemotherapy with immune checkpoint inhibitors has been reported to improve the clinical outcomes of patients with SCLC (2). Although early initiation of chemotherapy with immune checkpoint inhibitors (ICIs) is desirable to improve the prognosis and symptoms of patients with SCLC, there is concern that patients with SCLC and pericardial or pleural effusions may not be able to initiate chemotherapy safely due to poor performance status (PS). Moreover, it is unclear whether pleural/pericardial drainage and pleurodesis/pericardiodesis should be performed before or after initiating chemotherapy should in patients with chemotherapy-sensitive SCLC. Here, we report a case of successful initiation of chemotherapy combined with ICI therapy in a patient with SCLC and pleural and pericardial effusions, after performing pleurodesis and pericardial drainage.

## Case report

**Figure 1:**
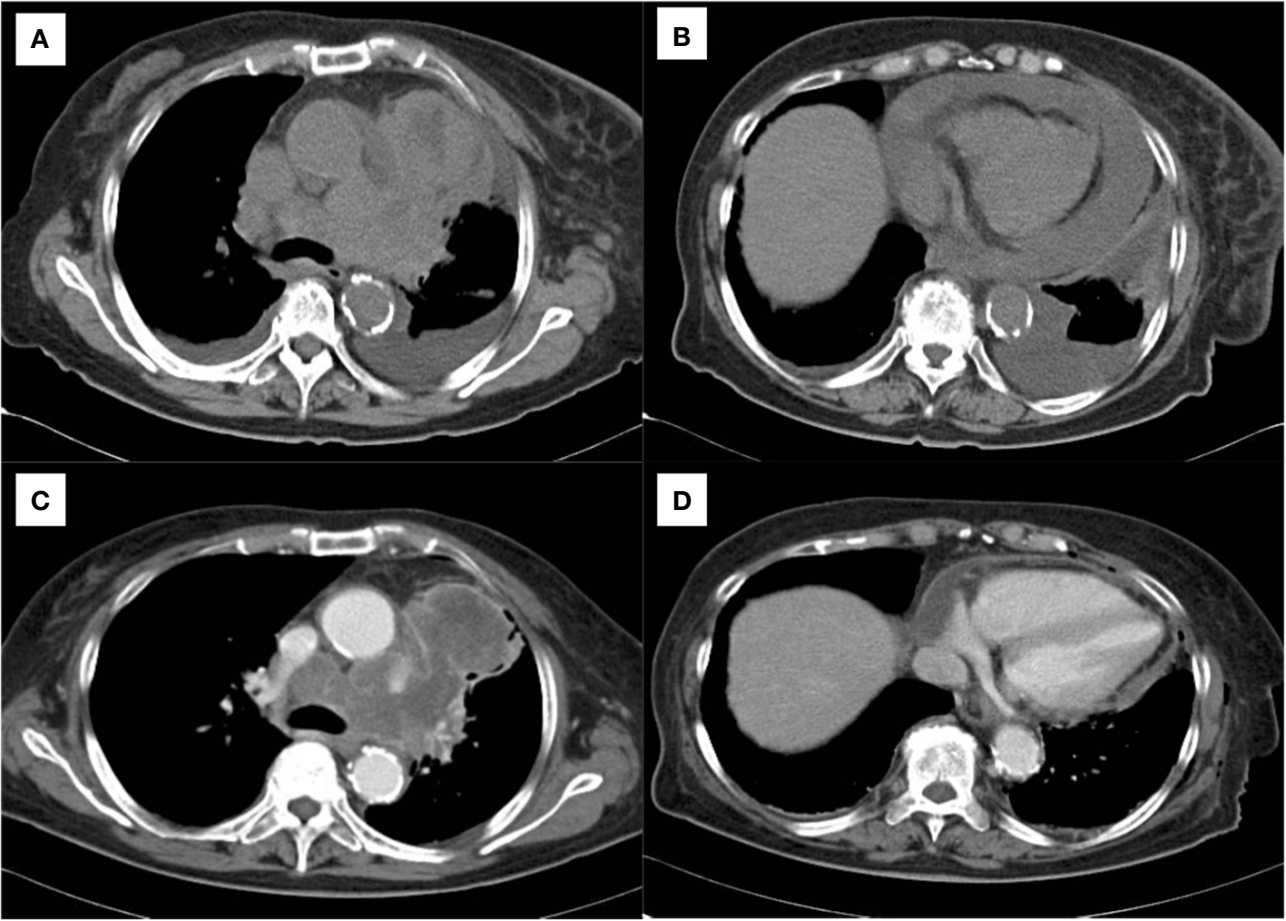
Chest computed tomography showing **(A)** primary lung cancer and bilateral pleural effusions and **(B)** pericardial effusion. **(C, D)** After drainage of the effusions and pleurodesis, showing control of the effusions before starting systemic chemotherapy.

**Figure 2:**
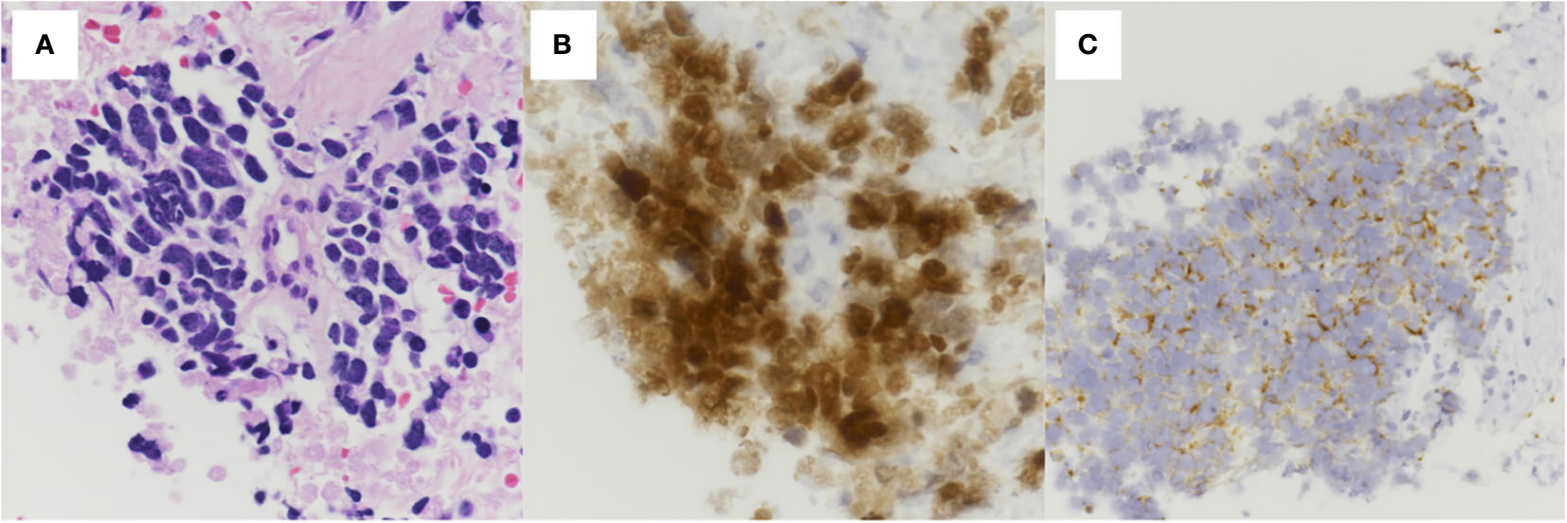
Histopathological findings of the pulmonary tumor. **(A)** Hematoxylin and eosin staining showing dense sheet-like growth of bare nucleated atypical cells with increased chromatin (400× magnification). Immunostaining showing positive **(B)** insulinoma-associated protein 1 (400× magnification) and **(C)** chromogranin A (200× magnification).

**Figure 3:**
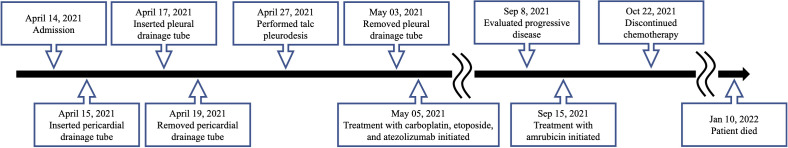
Timeline of the main interventions from the initial visit to the treatment course.

A 76-year-old woman presented to the emergency department with progressive dyspnea on exertion for a week. Her medical history included hypertension, dyslipidemia, and hyperuricemia. Her vital signs at the visit were: body temperature 36.1°C, pulse rate 68 beats/min, blood pressure 130/78 mmHg, respiratory rate 18 breaths/min, and oxygen saturation 92% breathing ambient air, and her consciousness was clear. Physical examination revealed decreased breath sounds in the lower left side of the chest and decreased cardiac sounds. Blood tests revealed white blood cell count 7,700/μL, aspartate aminotransferase 52 IU/L, alanine aminotransferase 55 IU/L, C-reactive protein 0.97 mg/dL, pro-gastrin-releasing peptide 71.9 pg/mL, and neuron specific enolase 254 ng/mL. Chest computed tomography (CT) showed a mass shadow anterior to the left upper lobe, bilateral pleural effusions (**Figure 1A**), and pericardial effusion circumferentially around the entire heart (**Figure 1B**). We performed pericardial drainage on day 2 because of concern about the possibility of the development of cardiac tamponade and for diagnosis. Pericardial fluid was exudative, but cytology of the fluid was negative for malignant cells. On day 4, the patient required oxygen therapy because of an increased left pleural effusion, and we performed pleural drainage. Pleural effusion was exudative, and cytology of the fluid was positive for small cell lung cancer. On day 8, we performed a CT-guided lung biopsy. Hematoxylin and eosin staining of the biopsy specimen revealed dense sheet-like growth of bare nucleated atypical cells with increased chromatin (**Figure 2A**), and immunostaining was positive for insulinoma-associated protein 1 (**Figure 2B**) and chromogranin A (**Figure 2C**) and negative for thyroid transcription factor-1, p40, and leukocyte common antigen (not shown). We diagnosed extensive-stage small cell lung cancer (ES-SCLC) based on the clinical course and pathological findings. The pleural fluid was drained over 5,000ml and we confirmed lung re-expansion on chest X-ray. Later, we performed talc pleurodesis to control the pleural effusion (**Figures 1C, D**) and improve the PS, which was 2 at the initial visit, and subsequently improved to 0. We did not perform pericardiodesis due to concern about the risk of adverse events. Instead, we administered a combination regimen of carboplatin, etoposide, and atezolizumab as first-line chemotherapy. After 4 cycles of first-line chemotherapy, we evaluated the patient to have progressive disease, so we administered amrubicin as second-line chemotherapy. However, her general condition worsened after 2 cycles, and considering her wishes, she was treated with best supportive care alone after that. The patient died approximately 9 months after diagnosis. The timeline of all relevant interventions from the initial visit to the introduction of treatment is shown in **Figure 3**.

## Discussion

Based on the TNM classification, the 5-year survival rate for patients with SCLC with T4 lesions and pleural effusions is 3.6%, with a median overall survival of 7 months (3). Patients with ES-SCLC with pleural effusion on the same side as the primary tumor have a significantly lower overall survival rate than ES-SCLC without pleural effusion (20.9 months vs. 11.8 months, p < 0.001) (4). In patients with SCLC, the presence of malignant pleural effusions is an independent prognostic factor for SCLC, and reduce the median overall survival time by approximately 4 months, and are associated with lower 1-year and 2-year survival rates (17% and 6%, respectively) (5). The median overall survival of patients with SCLC and pericardial effusions is 14.2 months, which is approximately 7 months shorter than that of SCLC patients without pericardial effusions (1). In addition, metastasis of SCLC to the pericardium generally occurs at a relatively late stage in the disease (6). Therefore, the early initiation of chemotherapy is desirable in patients with SCLC patients and pleural effusions, pericardial effusions, or both.

In the IMpower133 trial, which tested the efficacy of a regimen combining atezolizumab, carboplatin, and etoposide compared to placebo, combination chemotherapy increased the overall survival by 2 months (12.3 months vs. 10.3 months, p < 0.01), with no increase in serious adverse events compared to standard therapy (2). In addition, ICIs, including pembrolizumab and durvalumab, combined with cytotoxic anticancer agents improve progression-free survival and overall survival (7, 8). Thus, early initiation of chemotherapy with ICIs, especially in patients with SCLC and pleural or pericardial effusions, who generally have a poor prognosis (9), may contribute to improving the clinical outcome.

A prospective observational study with malignant pleural effusion control as an outcome, conducted in cancer patients with pharmacologically sensitive and non-pharmacologically sensitive tumors of whom 13% had SCLC, found that factor that had the greatest effect on controlling malignant pleural effusions was not whether the patients received systemic chemotherapy, but whether they underwent pleurodesis (10). Another prospective observational study of 509 patients with lung cancer and malignant pleural effusions found that compared to chemotherapy alone, early treatment of malignant pleural effusions reduced the need for future re-intervention (23.5% vs. 53.8%, p < 0.01) (11). In patients with non-small cell lung cancer, intrapericardial chemotherapy alone and a combination of intrapericardial chemotherapy and systemic chemotherapy improve control of cancer-related pericarditis (12–14). These results suggest that in patients with SCLC and pleural and pericardial effusions, drainage and pleurodesis/pericardiodesis prior to chemotherapy might lead to better fluid control and improved symptom control and prognosis.

In this case, we were concerned about the increased risk of adverse events with combined pleurodesis and pericardiodesis; therefore, we performed pleurodesis and pericardial drainage, without pericardiodesis. As a result, chemotherapy could be safely introduced with improved PS. However, there are several limitations in this case. First, the clinical course of chemotherapy preceded by pericardial or pleural drainage is uncertain. Randomized controlled trials of non-small cell lung cancer with pleural effusion suggested that a pleural effusion control rate of 86.9-92.9% may be achieved with chemotherapy alone without pleurodesis (15, 16). Local approaches, such as drainage to the pericardial and thoracic space, are unlikely to result in serious outcomes (12, 17) but may delay the administration of chemotherapy in about 2% of patients (18). Second, the patient’s clinical response was not adequate. The patient had to stop chemotherapy during the second round of chemotherapy due to poor performance status, resulting in death 9 months after diagnosis. In other words, the clinical course could have been different if chemotherapy had been administered prior to the procedure. Third, the impact of pleurodesis on PET/CT, which may be performed to determine the efficacy of chemotherapy, needs to be considered (19). In this patient, PET/CT was not performed due to the inaccessibility and financial burden of PET/CT. We needed to plan our treatment strategy with these matters in mind carefully.

This case highlights the importance of considering aggressive drainage and pleurodesis/pericardiodesis before systemic chemotherapy in patients with SCLC and concurrent pericardial or pleural effusions, even in patients with SCLC that is sensitive to chemotherapy.

## Patient perspective

I went to the hospital because I was short of breath, and had fluid drained from around my heart and lungs. This treatment was successful, and I was able to receive chemotherapy before the cancer worsened. Fortunately, I did not experience any major side effects of chemotherapy, and I am continuing to live my daily life.

## Data availability statement

The raw data supporting the conclusions of this article will be made available by the authors, without undue reservation.

## Ethics statement

Ethical review and approval was not required for the study on human participants in accordance with the local legislation and institutional requirements. The patients/participants provided their written informed consent to participate in this study.

## Author contributions

AK, YF, MS, and MI collected and interpreted the clinical data. AK and YF drafted the manuscript. MS, MI, and HS reviewed and revised the manuscript. All authors read and approved the final version of the manuscript.
